# Assessment of knowledge and practices of exclusive breastfeeding among rural women during the COVID-19 pandemic in Egypt: a cross sectional study

**DOI:** 10.1186/s12905-023-02831-0

**Published:** 2023-12-18

**Authors:** Noura El-Gamel, Amina El-Nemer

**Affiliations:** Woman’ s Health and Midwifery Nursing, Faculty of Nursing, Mansoura University, Mansoura, Egypt

**Keywords:** COVID-19, Exclusive breastfeeding, Knowledge, Practices, Rural women

## Abstract

**Background:**

Worldwide exclusive breastfeeding is still recommended as a successful strategy even during the COVID -19 pandemic to lower infant morbidity and mortality. This study aimed to assess the knowledge and practices of exclusive breastfeeding among rural women during the COVID-19 pandemic.

**Methods:**

A descriptive cross-sectional study was conducted at EL-Morabeen Family Medicine Center in rural Damietta, Egypt among 178 lactating women who were chosen by using the purposive sampling technique. A developed structured questionnaire consisting of four parts was used to gather data from March to May 2022. Univariate analysis for descriptive data and bivariate analysis through the chi-square test were performed.

**Results:**

The current study revealed that 73% of the studied rural women did not receive any breastfeeding counseling during antenatal visits and 61.2% of them believed that coronavirus was transmitted through breastmilk. Only 15.2% of them breastfed their infant exclusively for 6 months, 88.2% of mothers delayed breastfeeding initiation after delivery and 48.3% administered the prelacteal feeds. A total of 98.3% of rural women had never made skin-to-skin contact, and 79.2% of them had not been vaccinated against COVID-19. Additionally, a statistically significant association between good knowledge and practice with highly educated women aged 26–30 years, with a monthly income of 4000–6000 L.E was found. Furthermore, only 26.4% and 26.1% of rural women had good knowledge and practice scores respectively.

**Conclusion:**

Suboptimal breastfeeding practices, such as delayed onset of breastfeeding, low percentages of exclusivity, early weaning, prelacteal feeding administration, and lack of skin-to-skin contact during the COVID -19 pandemic were prevalent among the studied rural mothers. Breastfeeding counseling for all pregnant women and implementation of evidence-based practices in the health care system, such as the early initiation of breastfeeding and skin-to-skin contact, are recommended.

## Background

Providing breast milk only to newborn infants except for supplements or medications during the 1st six months of life is known as exclusive breastfeeding (EBF) [1]**.** EBF is the clinical gold standard for infant feeding. It grants unique health benefits for infants and mothers. Breast milk is designed specifically to satisfy the health needs of a growing newborn [2]**.** Colostrum is recognized as the first infant vaccine and a powerful natural medication. It has significant levels of antibodies that defend against communicable and infectious diseases [3, 4]**.**

Optimal breastfeeding practices include initiating lactation during the first hour after birth, rooming-in, lactating exclusively on demand, continued along with nutritionally adequate and safe complementary food until the age of two years are the ideal newborn feeding strategy for promoting infants’ healthy growth and development [5]**.** Optimal practices could save the lives of 820 000 children under the age of 5 years annually, raise the intelligence quotient (IQ) from 3 to 4 points, increase school attendance, and prevent approximately 20,000 breast cancer deaths. Optimal practices enable the nation to save hundreds of millions of dollars spent on health care by promoting child development and reducing healthcare costs [6]**.**

Exclusive breastfeeding rates were poor, especially in developing countries, and it took over a decade to increase from 33 to 39%. Nearly 60% of the world’s infants are missing out on the recommended six months of EBF. In 2018 only 43% of babies worldwide were breastfed within the first hour of their life [7]**.** In Egypt, despite significant efforts over the last few decades to reduce infant and child mortality, chronic malnutrition among children under the age of five remains a major problem throughout the country, with stunting increasing from 23% in 2005 to 29% in 2008 [8].

However, breastfeeding is a common practice in Egyptian culture. The 2014 Egypt Demographic and Health Survey found that only 27% of women started lactation during the first hour after birth compared to 52% in 2008. Additionally, 13% of mothers breastfeed their infants exclusively for up to four or five months, compared to 29% in 2008. Incorrect EBF practices and pre-lacteal feeding are common in Egyptian culture and are associated with childhood malnutrition [9]**.**

Before the COVID-19 pandemic, several studies had shown a lack of knowledge regarding EBF and suboptimal breastfeeding practices among rural mothers. Therefore, the CDC recommends efforts to promote breastfeeding particularly focusing on rural mothers as one of the priority categories [10–13]**.** During the COVID-19 pandemic which has become a new obstacle for the health care system worldwide and continuity of health practices, the WHO recommended early breastfeeding, skin-to-skin contact (SSC) and EBF among all mothers regardless of their confirmed COVID-19 status. The WHO endorsed the use of protective measures before and after infant contact. As there is no proof of COVID transmission by breastfeeding [8]**.** However, clear WHO recommendations regarding EBF practice during the pandemic, infected mothers delayed the initiation or even expression of breast milk until the third week after birth because of quarantine measures [14]**.**

Early in the pandemic, some countries adopted nonevidence-based procedures such as infant-mother separation and stopping breastfeeding for suspected cases. In addition to the limited social contact, community breastfeeding support groups were not accessible to parents in need of assistance [15]**.** Moreover, several countries have reported that producers of infant formula have promoted it as a safer alternative to breastfeeding during the COVID-19 pandemic [14]**.** Physical distancing rules also led to fewer contact with mothers resulting in fewer opportunities for effective breastfeeding support.

During the COVID-19 pandemic, there was inaccurate information and widespread misconceptions regarding coronavirus transmission through breast milk resulting in suboptimal practices such as reducing the duration or cessation of breastfeeding. In Egypt, to the best of our knowledge, no data are available about rural women’s knowledge and practices regarding EBF during the pandemic. Hence, this study aimed to assess the knowledge and practices of exclusive breastfeeding among rural women during the COVID-19 pandemic to add baseline data on the EBF situation in Egypt.

## Methods

### Study design

A descriptive cross sectional study design was used to assess the knowledge and practices of exclusive breastfeeding among rural women during the COVID-19 pandemic.

### Study setting

The study was conducted at El-Morabeen Family Medicine Center in rural Damietta governorate, Egypt. The study setting includes two vaccination clinics for infants and children that are accessible two days a week (Saturdays and Tuesdays from 8 AM to 1 PM).

### Sampling technique

This study used a nonprobability purposive sample of 178 lactating women who attended the Family Medicine Center. They were selected according to the study’s inclusion and exclusion criteria.

#### Inclusion criteria

1. Breastfeeding rural mothers.
2. Mothers with infants aged from 0 to 6 months.

#### Exclusion criteria

1. Infants with birth defects that may affect breastfeeding such as cleft lip or cleft palate.
2. Preterm infants were separated from mothers during the first hour after delivery.
3. Mothers who have mental or psychological disorders.

### Sample size

Based on data from the literature [16]**,** considering that the power of the study is 80%, with a precision/absolute error of 5% and type 1 error of 5%, the sample size was calculated according to the following equation: Sample size = [Z_1-α/2_)^2^. P(1-P)]/d^2^ Whereas, ^Z^_1-α/2_ = is the standard normal variate, at 5% type 1 error (p < 0.05), it is 1.96. P = the expected proportion in the population based on previous studies. d = absolute error or precision. Therefore, the Sample size = [(1.96)^2^. (0.347). (1–0.347]/ (0.07)^2^ = 177.6. So, the needed sample is 178.

### Study tool

Data were gathered by the researcher using a structured questionnaire developed after reviewing the relevant literature [3, 15, 17, 18]**.** It consisted of four parts. 

#### Part one

Demographic traits of rural women that included age, level of education, occupation, and family income. 

#### Part two

Obstetric history that included gravidity, parity, gestational age, and mode of previous delivery. 

#### Part three

Exclusive breastfeeding knowledge of rural women during the COVID -19 pandemic. It consisted of 17 questions such as the definition of EBF, the optimal duration of EBF, infant benefits of EBF, maternal benefits of EBF, and breastfeeding recommendations during the COVID -19 pandemic (i.e., the use of standard precautions, WHO recommendations for breastfeeding in case of infection with coronavirus).

### Knowledge scoring system

Each question had two alternative answers: correct and incorrect. The responder score was 1: 0 for each response. The total knowledge score was calculated based on the number of questions answered in which more than 75% considered good knowledge,50–75% considered fair knowledge, and less than 50% considered poor knowledge [19]**.**

#### Part four

Exclusive breastfeeding practices of rural women during the COVID -19 pandemic. It consisted of 16 questions such as initiation time, frequency of feeding, prelacteal feeding, COVID vaccination state, and performance of respiratory hygiene during the pandemic. 

### Practice scoring system

Each question had two alternative answers: yes, and no. The responder score was 1: 0 for each response. The total practice score was calculated based on the number of questions answered with more than 75% considered good practice, 50–75% considered fair practice and less than 50% considered poor practice [19]**.**

### Data quality control

The validity of the study tool was evaluated by three experts in woman’s health and midwifery nursing at the faculty of nursing -Mansoura university. The reliability of the study tool was tested by Cronbach's alpha. The Cronbach’s alpha value (internal consistency) in the knowledge section was 0.874, and that in the practice section was 0.902.

### Pilot study phase

After designing the tool, a pilot study including 18 women who met the study criteria and represented 10% of the total sample was carried out in the same setting to assess the clarity, and applicability of the tool and any obstacles in collecting the data. The pilot participants were eliminated from the study sample. This step took a month (February 2022).

### Fieldwork

Data were gathered over a three -month period beginning in March 2022 and ending in May 2022.The researcher attended two days a week (Saturday and Tuesday) from 8 a.m. to 1 p.m. After self-introduction to the nurses and the mothers, the researcher interviewed the mothers to choose only participants who met the inclusion criteria of the study. Then the researcher explained the study’s aim and obtained the mothers’ informed written consent to participate in the study. Each mother was interviewed individually for 15 to 20 minutes to gather data via a structured questionnaire. The researcher read each question to the woman and explained its meaning in Arabic before recording her response.

### Statistical analysis

The Statistical Package for Social Sciences (SPSS) version 20 was used to analyze the gathered data. Cronbach’s alpha was used to test the internal consistency of the study tool. Descriptive statistics such as number, percentage, mean, and standard deviation (mean ± SD) were utilized for quantitative data. The chi-square test was used to detect the association between categorical variables. At a *p* value of ≤ 0.05, the association was statistically significant, and at a *p* value of < 0.001, it was highly statistically significant. Finally, the results are presented in tables and figures.

## Results

Table 1 reveals that the average age of the studied women was 27.4 ± (4.3). Nearly half (48.3%) of them had a university education or higher. Additionally, more than half (55.6%) of them were housewives. More than three-quarters (80.9%) of family income ranged between 4000 and < 6000 L.E.

**Table 1:** Demographic characteristics of the studied rural women

Variables	(*n* = 178)	%
Age (years)		
≤ 20	18	10.1
21 – 25	41	23.1
26 – 30	91	**51.1**
> 30	28	15.7
Mean ± SD	**27.4 ± 4.3**	
Education		
Secondary Education	59	33.2
Institution	33	18.5
University or higher	86	**48.3**
Occupation		
Working	79	44.4
Housewife	99	**55.6**
Monthly income (L.E.)		
< 4000	27	15.2
4000 – < 6000	144	**80.9**
6000 – 10,000	7	3.9

Table 2 reveals that nearly three-quarters (74.2% and 76.4%) of the studied women were multigravida 2–3 times and had parity from two to three times. Moreover, more than two- thirds (71.9%) of them had C.S deliveries. Additionally, most (92.1%) of the studied women delivered at term. Also, the majority (85.4%) of infant birth weights were within the normal range.

**Table 2:** Obstetric history of the studied rural women

Variables	(*n* = 178)	%
Gravidity		
1	13	7.3
2 – 3	132	**74.2**
More than 3	33	18.5
Parity		
1	14	7.9
2 – 3	136	**76.4**
More than 3	28	15.7
Abortions		
None	168	**94.4**
Once	8	4.4
2 – 3	1	0.6
More than 3	1	0.6
Living Children		
1	15	8.4
2 or More	163	**91.6**
Previous mode of delivery		
Cesarean Section	128	**71.9**
Vaginal Delivery	50	28.1
Gestational age (Weeks)		
Less than 37	11	6.2
37 – 42	164	**92.1**
More than 42	3	1.7
Newborn birth weight (K.G.)		
< 2.5	11	6.2
2.5 – 3.5	152	**85.4**
> 3.5	15	8.4
Age of youngest infant (months)		
< 2	1	0.6
2 – < 4	76	42.7
4 – 6	101	**56.7**

**Fig. 1:**
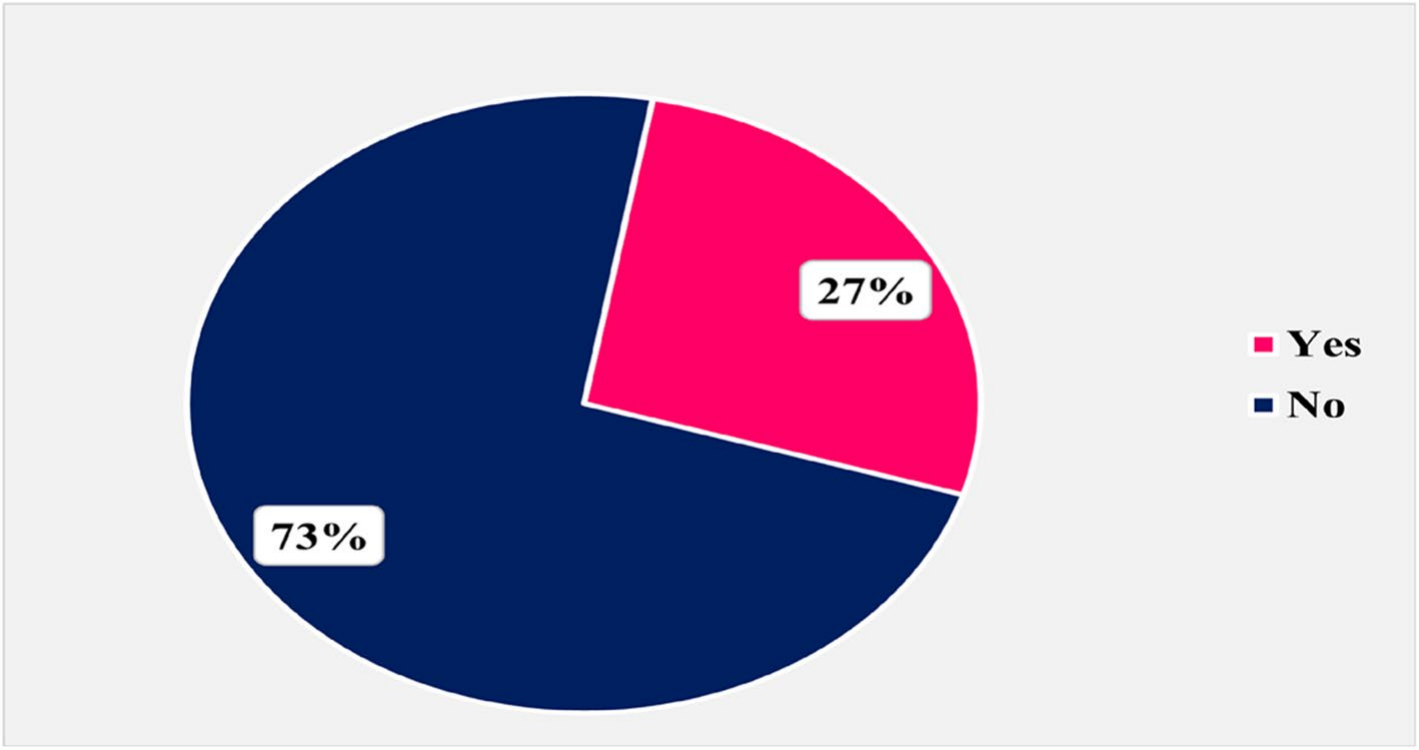
Percent of the studied rural women who received breastfeeding counseling during antenatal visits from a health care provider (*n* = 178)

Figure 1 shows that only 27% of the studied rural women received breastfeeding counseling from a health care provider.

Table 3 shows that three-quarters (75.8%) of the studied women could not define EBF correctly. Additionally, more than half (53.4%) of them did not know the best time for BF initiation. Nearly three- quarters (74.2%) of them were unaware of the recommended fluids for infants under the age of six months. In addition, more than two-thirds (70.2%) of them did not know the optimal age to start complementary food. Less than two-thirds (61.2%) of the studied women had incorrect ideas about the coronavirus transition through breastmilk, while more than half of them (53.9%) had incorrect information about recommending the COVID-19 vaccination for breastfeeding women.

**Table 3:** Exclusive breastfeeding knowledge among the studied rural women during the COVID-19 pandemic (*n* = 178)

**Variables**	**Correct**		**Incorrect**	
	n	%	n	%
Heard of exclusive breastfeeding	11	6.2	167	**93.8**
Exclusive breastfeeding definition	43	24.2	135	**75.8**
Best time to start breastfeeding	83	46.6	95	**53.4**
Colostrum benefits the baby	139	**78.1**	39	21.9
EBF protects newborns against infectious diseases	144	**80.9**	34	19.1
EBF protects newborns against chronic diseases	66	37.1	112	**62.9**
EBF protects women against breast and ovarian cancers	141	**79.2**	37	20.8
EBF protects women from certain chronic diseases	36	20.2	142	**79.8**
Frequency of breastfeeding	114	**64.0**	64	36.0
Recommended fluids for infants < 6 months	46	25.8	132	**74.2**
The optimal age to start complementary food	53	29.8	125	**70.2**
Management of scanty milk in the first 3 days	129	**72.5**	49	27.5
Coronavirus is transmitted by breastmilk	69	38.8	109	**61.2**
A breastfeeding mother can protect herself and infant from COVID-19 by				
• Maintaining a social distance of 1 m	174	**97.8**	4	2.2
• Avoiding contact with ill people	177	**99.4**	1	0.6
• Wearing a surgical mask outdoors	177	**99.4**	1	0.6
• Maintaining handwashing before and after infant contact	178	**100.0**	0	0.0
• Using hand sanitizers as alcohol	160	**89.9**	18	10.1
• Maintaining respiratory hygiene practices	161	**90.4**	17	9.6
WHO recommendations for COVID -19 suspected or positive mothers	106	**59.6**	72	40.4
WHO recommendations for COVID -19 severe positive mothers	108	**60.7**	70	39.3
COVID-19 vaccination recommended for breastfeeding women	82	46.1	96	**53.9**

**Fig. 2:**
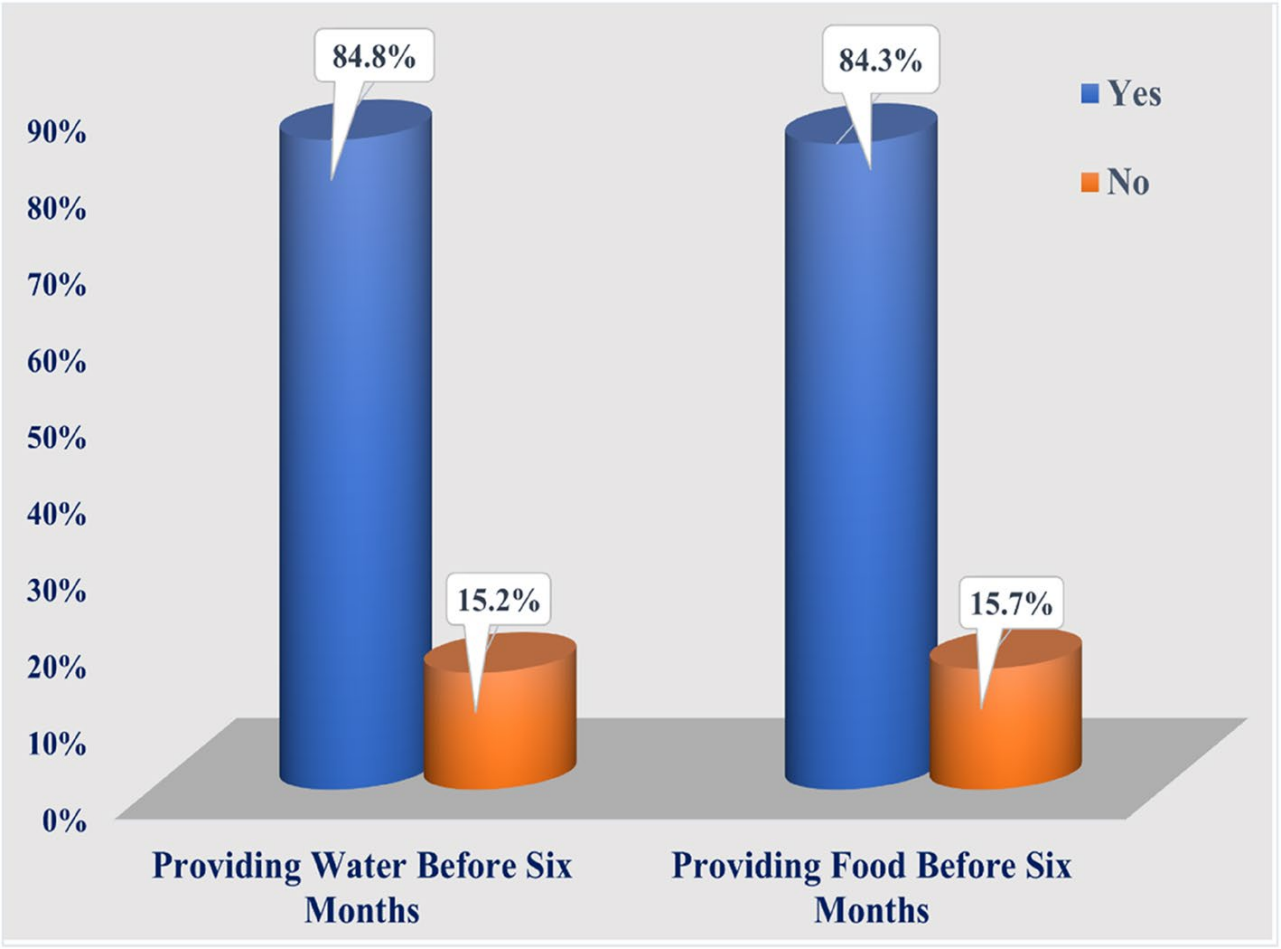
Water and food administration before the age of six months (*n* = 178)

Figure 2 shows that the majority (84.8% and 84.3%, respectively) of the studied rural women administered water and food to their infants before the age of six months.

Table 4 shows that the majority (88.2%) of the studied women did not initiate breastfeeding within the 1st hour after birth, while nearly half (48.3%) of them gave prelacteal feeds to their infants. In addition, the feeding duration was less than 15 min among more than two-thirds (41%) of women. The majority (84.8%) of the studied women did not breastfeed exclusively, as the majority (84.8% and 84.3%, respectively) of them provided water and food during the 1st six months. Moreover, most (98.3%) of the studied women did not practice skin-to-skin contact during the COVID-19 pandemic. Additionally, more than three-fourths (79.2%) of the studied women did not get vaccinated against coronavirus. However, two-thirds (66.3%) of the studied women maintained good respiratory hygiene.

**Table 4:** Exclusive breastfeeding practices of the studied rural women during the COVID-19 pandemic (*n* = 178)

Variables	Yes		No	
	N	%	N	%
Starting breastfeeding during the 1st hour after delivery	21	11.8	157	**88.2**
Feeding colostrum for the 1st 3 days	169	**94.9**	9	5.1
Giving prelacteal feeds to the newborn infant	86	**48.3**	92	51.7
Each feeding duration for a ≥ 15 min	105	59.0	73	**41.0**
Providing both breasts on each feed	46	25.8	132	**74.2**
Starting with last breast on the subsequent feed	68	38.2	110	**61.8**
Feeding only breast milk for the 1st six months	27	15.2	151	**84.8**
Using artificial teats or pacifiers	163	**91.6**	15	8.4
Practicing skin-to-skin contact	3	1.7	175	**98.3**
Allowing others to kiss the infant	172	**96.6**	6	3.4
Vaccinated against the coronavirus	37	20.8	141	79.2
Maintain good respiratory hygiene	118	**66.3**	60	33.7
Committed with facemask outdoors	100	**56.2**	78	43.8
Washing hands after coughing or sneezing	101	**56.7**	77	43.3

**Fig. 3:**
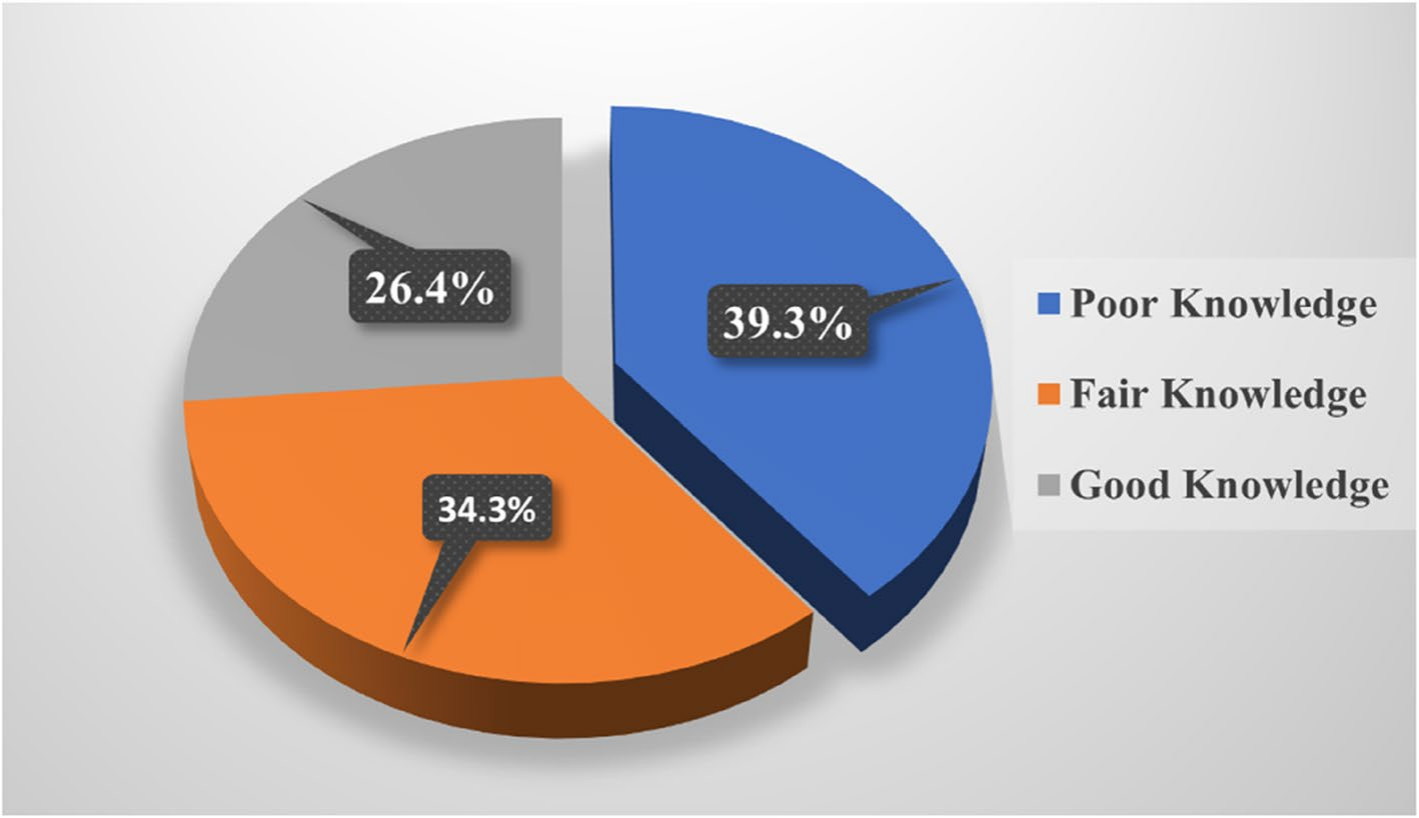
Total exclusive breastfeeding knowledge score among the studied rural women during the COVID-19 pandemic (*n* = 178)

Figure 3 shows that nearly one-fifth (39.3%) of the studied rural women had poor knowledge of exclusive breastfeeding during the pandemic.

**Fig. 4:**
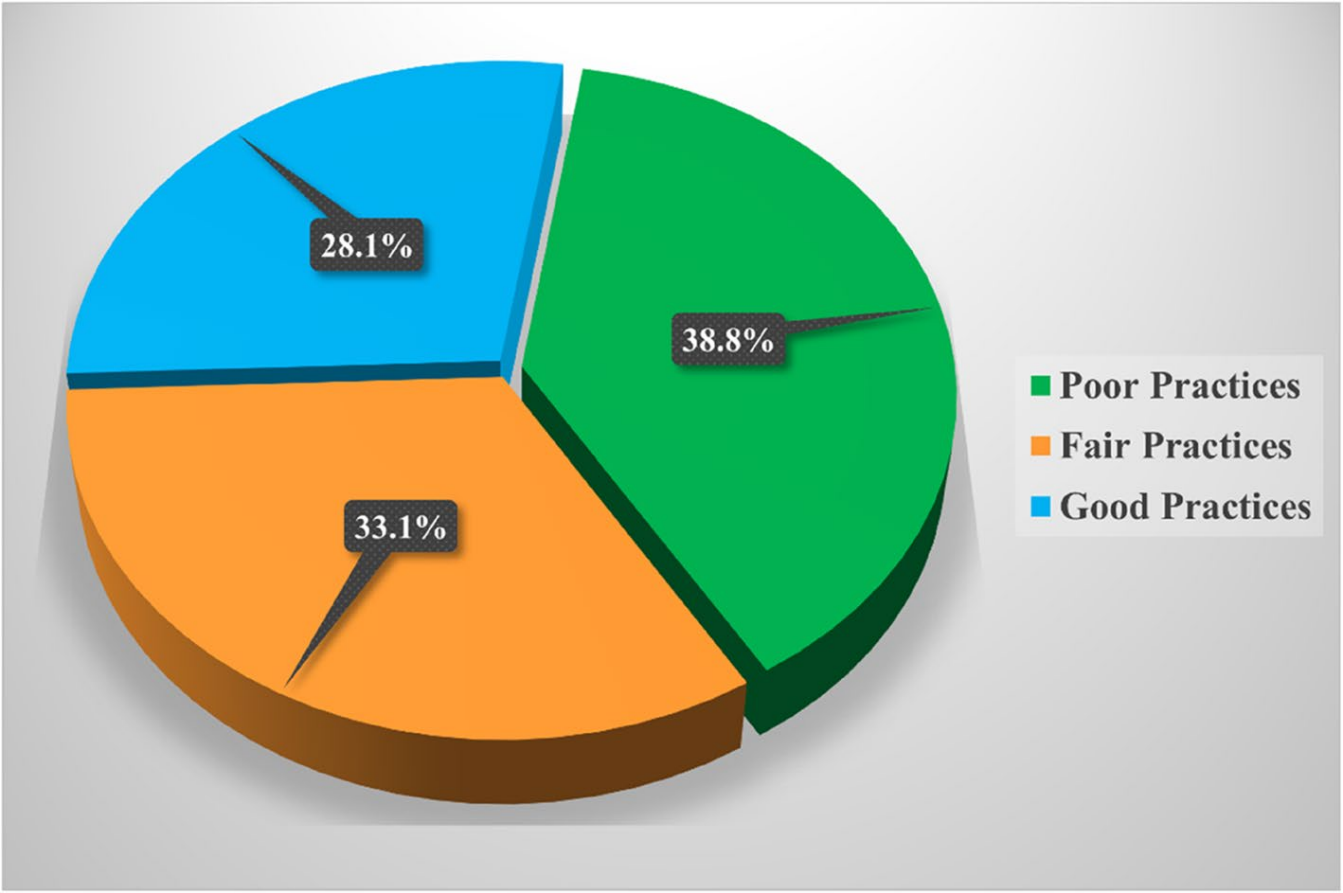
Total exclusive breastfeeding practice score among the studied rural women during the COVID-19 pandemic (*n* = 178)

Figure 4 shows that nearly one-fifth (38.8%) of the studied rural women had poor practices of exclusive breastfeeding during the pandemic.

Table 5 reveals a highly statistically significant association between the total knowledge score and age, educational level, and occupation (*P* < 0.001). Additionally, there was a statistically significant relationship between knowledge score and family income (*P* < 0.006).

**Table 5:** Association between demographic characteristics of the studied rural women and exclusive breastfeeding knowledge during the COVID-19 pandemic (*n* = 178)

Variables	Poor Knowledge (*n* = 70)		Fair Knowledge (*n* = 61)		Good Knowledge (*n* = 47)		Significance test	
	N	%	N	%	N	%	*X*^*2*^	*P*
**Age (years)**								
≤ 20	16	22.9	2	3.3	0	0.0		
21 – 25	27	38.6	9	14.8	5	10.6		
26 – 30	12	17.1	40	65.6	39	**83.0**		
> 30	15	21.4	10	16.4	3	6.4	62.804	< 0.001**
**Education**								
Secondary education	52	74.3	7	11.5	0	0.0		
Institution	15	21.4	15	24.6	3	6.4		
University or higher	3	4.3	39	63.9	44	**93.6**	116.361	< 0.001**
**Occupation**								
Working	19	27.1	30	49.2	30	**63.8**		
Housewife	51	72.9	31	50.8	17	36.2	16.198	< 0.001**
**Monthly income (L.E.)**								
< 4000	17	24.3	9	14.8	1	2.1		
4000 – < 6000	49	70.0	49	80.3	46	**97.9**		
6000 – 10,000	4	5.7	3	4.9	0	0.0	14.380	0.006^*^

χ2: Chi -square test

*(P) Significant at *P* ≤ 0.05

**High significant at *P* ≤ 0.001

Table 6 reveals a highly statistically significant positive relationship between practices score, age, and educational level(*P* < 0.001). In addition, there was a statistically significant positive relationship regarding occupation (*P* = 0.008) and family income (*P* = 0.013).

**Table 6:** Association between demographic characteristics of the studied rural women and exclusive breastfeeding practices during the COVID-19 pandemic (*n* = 178)

Variables	Poor Practices (*n* = 69)		Fair Practices (*n* = 59)		Good Practices (*n* = 50)		Significance test	
	N	%	N	%	N	%	X^2^	P
**Age (years)**								
≤ 20	14	20.3	4	6.8	0	0.0		
21 – 25	26	37.7	10	16.9	5	10.0		
26 – 30	10	14.5	41	69.5	40	**80.0**		
> 30	19	27.5	4	6.8	5	10.0	64.156	< 0.001**
**Education**								
Secondary education	48	69.6	11	18.6	0	0.0		
Institution	17	24.6	15	25.4	1	2.0		
University or higher	4	5.8	33	55.9	49	**98.0**	110.270	< 0.001**
**Occupation**								
Working	21	30.4	29	49.2	29	**58.0**		
Housewife	48	69.6	30	50.8	21	42.0	9.738	0.008^*^
**Monthly income (L.E.)**								
< 4000	18	26.1	6	10.2	3	6.0		
4000 – < 6000	47	68.1	51	86.4	46	**92.0**		
6000 – 10,000	4	5.8	2	3.4	1	2.0	12.674	0.013^*^

χ2: Chi -square test

*(P) Significant at *P* ≤ 0.05

** High significant at *P *≤ 0.001

## Discussion

The current study surveyed the knowledge and practices of rural women regarding exclusive breastfeeding during the COVID-19 pandemic and found a statistically positive link between knowledge, practice and highly educated, working mothers with family incomes ranging from 4000–6000 L.E. per month. These findings were supported by several Egyptian studies conducted by [13] in upper Egypt [20], in Mansoura**, **[21] in Giza, who found a significant relationship between good knowledge, practice scores and demographic data.

### Rural women’s knowledge of EBF during the COVID-19 pandemic

This study reported that only one-third of the studied mothers received breastfeeding counseling during antenatal visits from a health care provider, whereas it was higher in the study of [22] who found that three-quarters of the studied women received breastfeeding counseling during antenatal visits. This might be explained by different follow-up settings in both studies. This is a missed opportunity to counsel mothers about the value of exclusive breastfeeding for both the mother and infant’s health during antenatal care visits.

In addition, the current study found that EBF term was unfamiliar among most of the studied mothers, and three-quarters of them did not know the definition of EBF. This result was supported by the research conducted by [23] in Indonesia and [24] in Ghana, which concluded that most of the studied women did not hear of exclusive breastfeeding, and nearly one-quarter of them were unable to define EBF. Additionally, two-thirds of them incorrectly defined EBF.

Regarding COVID transmission through breast milk, this study showed that more than half of the studied women had the misconception that coronavirus could be transmitted through breastmilk. Similarly, a study conducted by [25] in India and [26] in Turkey reported that half of participants thought that COVID-19 is transmitted through breast milk. However, the study of [27] revealed that more than three quarters of the sample agreed that COVID-19 is transmitted through breastmilk.

During the COVID-19 pandemic, the WHO recommended a set of preventive measures for all populations, including breastfeeding women, to control the spread of the virus, which have been adopted by the Egyptian government to reduce the burden of COVID-19 in Egypt. As a result, the current study showed that most of the studied women had good knowledge of standard precautions (maintaining a social distance of 1 m, avoiding contact with ill people, wearing a mask, practicing handwashing, using sanitizers, and good respiratory hygiene).

These findings agreed with [28]**,** who revealed that most of the studied women in 5 countries had good knowledge of hygienic practices. Also [29], study in Nigeria revealed that the majority of the studied women had good knowledge. Conversely, a study in Bangladesh conducted by [30] showed that only half of the respondents identified standard precautions. The study of [30] explained his result by several factors, such as low socioeconomic status among the studied sample and the need for more female education.

### Rural women’s practice of EBF during the COVID-19 pandemic

The WHO classifies the rate of early initiation of breastfeeding as poor if it equals from 0 to 29%, as fair if it equals from 30 to 49%, as good if it equals from 50 to 89%, and as very good if it equals from 90 to 100% [31]**.** The current study found the early initiation rate to be poor according to the WHO classification. This finding was consistent with other Egyptian studies [22] that found a 5.5% prevalence of early initiation, [32] which showed that the prevalence of early initiation was 2.7%. In contrast, this result was much lower than the study of [33]**,** which reported the breastfeeding initiation was 27% within the first hour after delivery.

Additionally, the current study revealed that the EBF rate for the first 6 months was only 15.2%. This is far from the WHO target level of achieving a 50% exclusive breastfeeding rate worldwide. Additionally, this finding was lower than the results of [22]**,** who found that 28% of infants were exclusively breastfed; [12] revealed that EBF was 40%; 42.8% in the study conducted by [34] in Kampala Uganda; and a study in Iran conducted by [35]**,** showed that EBF was more than 50%.This disparity might be attributed to a knowledge gap regarding the duration and benefits of exclusive breastfeeding among the rural women included in our study.

Moreover, this study indicated that slightly more than half of the sample already gave prelacteal feeds. This was similar to several Egyptian studies [21] in Giza, Egypt found 53.2% of the studied women administered prelacteal feed, and [36] in Mansoura, Egypt, who reported that more than half 58% of newborns received prelacteal feeds. This may be due to prevalent myths about inadequate milk supply in the first three days following delivery in Egyptian culture. Also, the results are lower than the study findings of [37] in India, who found that slightly more than two-thirds of the studied women already gave prelacteal feeds.

However, early uninterrupted skin-to-skin contact is recommended by the WHO even during the pandemic to improve neonatal survival [38]**.** The current study showed that skin-to-skin contact is an uncommon practice by most of the studied women. This result was consistent with [39]**,** who revealed that only 10% of mothers reported SSC, and [40]**,** found that rates of SSC following a vaginal delivery were below 20% in low-income countries such as Tanzania, Ethiopia, and Nepal. Low rates could be explained by hospital policies that demand immediate mother-newborn separation. Additionally, there is a knowledge gap among the studied women about the skin-to-skin contact concept, and its benefits.

### Study limitations

The data were collected during the later stages of the pandemic, which suggests that women may have exhibited less caution toward COVID-19 and were not committed to standard precautions. Consequently, the findings of the study may vary from those obtained during the earlier stages of the pandemic.

## Conclusion

The findings of this study highlighted several key areas of concern and misconceptions regarding exclusive breastfeeding as believing in the importance of prelacteal feeds to newborns and COVID transmission through breast milk. Suboptimal breastfeeding practices such as delayed initiation of breastfeeding, low rates of exclusivity, and lack of skin-to-skin contact during COVID-19 were prevalent compared to WHO recommendations. Hence, breastfeeding counseling for all pregnant women and the implementation of evidence-based practices in maternity care, such as the early initiation of breastfeeding and skin-to-skin contact are recommended.

## Data Availability

The datasets used and analyzed during the current study are available from the corresponding author on reasonable request.

## Abbreviations

CDC: Centers for Disease Control and Prevention
COVID -19: Coronavirus
EBF: Exclusive Breastfeeding
IQ: Intelligence Quotient
SCC: Skin-to-Skin Contact
SD: Standard Deviation
SPSS: Statistical Package for Social Sciences
WHO: World Health Organization

## References

[1] World Health Organization (WHO). Infant and young child feeding. 2021. Available from: https://www.who.int/news-room/fact-sheets/detail/infant-andyoung-child-feeding.

[2] Center for disease control and prevention (CDC). Breastfeeding Is an Investment in Health. 2022 [cited 2022 October 21]. Available from: https://www.cdc.gov/breastfeeding/about-breastfeeding/why-it-matters.html

[3] Chowdhury FR, Yasmeen BN, Rahman S. Study on Exclusive Breastfeeding practice and related factors among mothers attending in a tertiary care hospital of Bangladesh. Northern Int. Med. Coll. J. [Internet]. 2018 Dec. 20 [cited 2022 Dec. 23];10(1):343–6. Available from: https://www.banglajol.info/index.php/NIMCJ/article/view/39329

[4] Pereira A, Cruz-Melguizo S, Adrien M, Fuentes L, Marin E, Forti A, et al. Breastfeeding mothers with covid-19 infection: A case series. International Breastfeeding Journal. 2020;15(1). Available from: 10.1186/s13006-020-00314-8 10.1186/s13006-020-00314-8 PMC7414278 32770999

[5] Latorre G, Martinelli D, Guida P, Masi E, De Benedictis R, Maggio L. Impact of covid-19 pandemic lockdown on exclusive breastfeeding in noninfected mothers. International Breastfeeding Journal. 2021;16(1). Available from: 10.1186/s13006-021-00382-4 10.1186/s13006-021-00382-4 PMC8052849 33865408

[6] Global Breastfeeding Collective (GBS). The Role of Midwives and Nurses in Protecting, Promoting and Supporting Breastfeeding [Internet]. 2019 [cited 2022 October 20]. Available from: https://www.globalbreastfeedingcollective.org/media/1391/file/GBC-advocacy-brief-role-midwives-nurses-protecting-promoting-breastfeeding.pdf

[7] Asare, B.YA., Preko, J.V., Baafi, D. et al. Breastfeeding practices and determinants of exclusive breastfeeding in a cross-sectional study at a child welfare clinic in Tema Manhean, Ghana. Int Breastfeed J 13, 12 (2018). 10.1186/s13006-018-0156-y. 10.1186/s13006-018-0156-y PMC5840768 29541153

[8] El-Shafie AM, Kasemy ZA, Omar ZA, Alkalash SH, Salama AA, Mahrous KS, et al. Prevalence of short stature and malnutrition among Egyptian primary school children and their coexistence with anemia. Italian Journal of Pediatrics. 2020;46(1). Available from: 10.1186/s13052-020-00855-y 10.1186/s13052-020-00855-y PMC7325115 32600418

[9] Ministry of Health and Population (MOHP). Egypt Demographic and Health Survey 2014. 2015 [cited 2022 July 25]. Available from: http://dhsprogram.com/pubs/pdf/FR302/FR302.pdf

[10] Aschbrenner K ., & Lubker Cornish D. Barriers to Breastfeeding among Rural Women in the United States, The University of Northern Iowa Journal of Research, Scholarship, and Creative Activity. 12, 2016–2017, ISSN: 1558–8769. Available from: https://universitas.uni.edu/volume-12-2016-2017/barriers-breastfeeding-among-rural-women-united-states

[11] Veeranki SP, Nishimura H, Krupp K, Gowda S, Arun A, Madhivanan P. Suboptimal breastfeeding practices among women in rural and low-resource settings: A study of women in Rural Mysore, India. Annals of Global Health. 2017;83(3–4):577. Available from: 10.1016/j.aogh.2017.10.012 10.1016/j.aogh.2017.10.012 29221531

[12] Savadogo LG, Ilboudo B, Kinda M. Exclusive breastfeeding practice and its factors in rural areas of Burkina Faso. Open Journal of Epidemiology. 2018;08(02):67–75. Available from: https://www.scirp.org/journal/paperinformation.aspx?paperid=84565

[13] Senosy SA, Saleh LH, Alareed HR. Exclusive breastfeeding knowledge, practices and determinants among mothers in rural areas, Egypt. International Journal of Community Medicine and Public Health. 2020;7(7):2443. Available from: https://www.researchgate.net/publication/342505662_Exclusive_breastfeeding_knowledge_practices_and_determinants_among_mothers_in_rural_areas_Egypt

[14] United Nations Children's Fund (UNICEF). Breastfeeding safely during the COVID-19 pandemic. 2020 [cited 2022 October 20]. Available from: https://www.unicef.org/turkmenistan/stories/breastfeeding-safely-during-covid-19-pandemic

[15] Peng S, Zhu H, Yang L, Cao L, Huang X, Dynes M, et al. A study of breastfeeding practices, SARS-COV-2 and its antibodies in the breast milk of mothers confirmed with covid-19. SSRN Electronic Journal. 2020. Available from: https://pubmed.ncbi.nlm.nih.gov/34013217/10.1016/j.lanwpc.2020.100045 PMC7654387 34013217

[16] Tadele N, Habta F, Akmel D, Deges E. Knowledge, attitude and practice towards exclusive breastfeeding among lactating mothers in Mizan Aman Town, southwestern Ethiopia: Descriptive cross-sectional study. International Breastfeeding Journal. 2016;11(1). Available from: https://pubmed.ncbi.nlm.nih.gov/26925156/10.1186/s13006-016-0062-0 PMC4769508 26925156

[17] Alamirew MW, Bayu NH, Tebeje NB, Kassa SF. Knowledge and attitude towards exclusive breast feeding among mothers attending antenatal and immunization Clinic at Dabat Health Center , Northwest Ethiopia : a cross-sectional institution based study. Hindawi Nurs Res Pract. 2017;2017:1–10. Available from: https://pubmed.ncbi.nlm.nih.gov/29312785/10.1155/2017/6561028 PMC5624135 29312785

[18] Nuampa S, Tilokskulchai F, Patil CL, Sinsuksai N, Phahuwatanakorn W. Factors related to exclusive breastfeeding in Thai adolescent mothers: Concept mapping approach. Maternal & Child Nutrition. 2018;15(2). Available from: 10.1111/mcn.12714 10.1111/mcn.12714 PMC7198966 30303630

[19] Baig M, Jameel T, Alzahrani SH, Mirza AA, Gazzaz ZJ, Ahmad T, et al. Predictors of misconceptions, knowledge, attitudes, and practices of covid-19 pandemic among a sample of Saudi population and its impact: A cross-sectional study. 2020. Available from: 10.1371/journal.pone.0243526 10.1371/journal.pone.0243526 PMC7725365 33296420

[20] Abusaad, F., Algilany, A. Predictors of Breastfed Mother's knowledge, Attitude and Practice during COVID-19 Pandemic. International Egyptian Journal of Nursing Sciences and Research, 2022; 2(2): 27–35. 10.21608/ejnsr.2022.212148. Available from: https://ejnsr.journals.ekb.eg/article_212148.html

[21] Kamal N, Ismael F, Abdelrehim M, El-Khateeb A. Breastfeeding practice and perception among women attending Primary Health Care Center in Giza, Egypt. Minia Journal of Medical Research. 2021;32(3):29–39. Available from: https://mjmr.journals.ekb.eg/article_241635.html

[22] Tollah Mostafa Farag H, Essam El-Din Mohamed Ammar N, Yahia El-Awady M. Prevalence of breastfeeding and factors affect its practice in women attending Primary Health Care Units in Cairo. Al-Azhar Medical Journal. 2020;49(4):2033–40. Available from: https://journals.ekb.eg/article_120658.html

[23] Diana R, Adi AC. Mother's knowledge, attitude, and practice of exclusive breastfeeding. Indian Journal of Public Health Research & Development. 2019;10(3):887. Available from: https://scholar.unair.ac.id/en/publications/mothers-knowledge-attitude-and-practice-of-exclusive-breastfeedin

[24] Mogre, V., Dery, M. & Gaa, P.K. Knowledge, attitudes and determinants of exclusive breastfeeding practice among Ghanaian rural lactating mothers. Int Breastfeed J 11, 12 (2016). 10.1186/s13006-016-0071-z. 10.1186/s13006-016-0071-z PMC4869336 27190546

[25] Thakur V, Kamal D, Swain S, Vikneshram CR. Knowledge, attitude, and practice toward COVID-19 among pregnant women in a tertiary care hospital during the COVID-19 outbreak. J Mar Med Soc. 2020;0(0):0. Available from: 10.4103/jmms.jmms_81_20

[26] Yassa M, Birol P, Yirmibes C, Usta C, Haydar A, Yassa A, et al. Near-term pregnant women’s attitude toward, concern about and knowledge of the COVID-19 pandemic. J Matern Fetal Neonatal Med. 2020;33(22):3827–34. Available from: 10.1080/14767058.2020.1763947 10.1080/14767058.2020.1763947 32429780

[27] Abuidhail J, Tamim F, Abdelrahman RY, Al-Shalabi E. Knowledge and practices of breastfeeding mothers toward prevention of the emerging corona virus (COVID- 19). Global Pediatrics. 2022;2:100024. 10.1016/j.gpeds.2022.100024. 37520671 10.1016/j.gpeds.2022.100024 PMC9479379

[28] Coca K, Lee EY, Chien L-Y, Souza ACDP, Kittikul P, Hong SA et al. Postnatal women’s breastfeeding beliefs, practices, and support during the COVID-19 pandemic: A cross-sectional comparative study across five countries. International breastfeeding journal. 2022 ;17(1):58. Epub 2022 Aug 17. 10.1186/s13006-022-00497-2 10.1186/s13006-022-00497-2 PMC9385077 35978362

[29] Anikwe CC, Ogah CO, Anikwe IH, Okorochukwu BC, Ikeoha CC. Coronavirus disease 2019: Knowledge, attitude, and practice of pregnant women in a tertiary hospital in Abakaliki, southeast Nigeria. Int J Gynecol Obstet. 2020;151(2):197–202. Available from: 10.1002/ijgo.13293 10.1002/ijgo.13293 PMC9087705 32608513

[30] Talukder A, Islam MN, Sarker M, et al. Knowledge and practices related to COVID-19 among mothers of under 2 children and adult males: a cross-sectional study in Bangladesh. BMJ Open. 2022;12:e059091. 10.1136/bmjopen-2021-059091. 35623761 10.1136/bmjopen-2021-059091 PMC9149685

[31] World Health Organization (WHO). Infant and young child feeding: a tool for assessing national practices, policies and programmes. 2003. Available from: https://apps.who.int/iris/handle/10665/42794

[32] Shaheen HM, Hegazy NN, Sakr SS. The barriers to breastfeeding among women: a single-center experience. Menoufia Med J. 2018;31:855–61. Available from: http://www.mmj.eg.net/text.asp?2018/31/3/855/248766

[33] Egypt demographic and health survey. Cairo, Egypt: Ministry of Health and Population and ICF International; 2015. 2014. Available from: https://dhsprogram.com/pubs/pdf/fr302/fr302.pdf

[34] Nabunya P, Mubeezi R, Awor P. Prevalence of exclusive breastfeeding among mothers in the informal sector, Kampala Uganda. PLOS ONE. 2020;15(9). Available from: 10.1371/journal.pone.0239062 10.1371/journal.pone.0239062 PMC7514031 32970700

[35] Behzadifar M, Saki M, Behzadifar M, Mardani M, Yari F, Ebrahimzadeh F, et al. Prevalence of exclusive breastfeeding practice in the first six months of life and its determinants in Iran: A systematic review and meta-analysis. BMC Pediatrics. 2019;19(1). Available from: 10.1186/s12887-019-1776-0 10.1186/s12887-019-1776-0 PMC6815441 31656169

[36] El-Gilany, A. H., & Abdel-Hady, D. M. Newborn first feed and prelacteal feeds in Mansoura, Egypt. BioMed research international, 2014.‏ Available from: https://www.hindawi.com/journals/bmri/2014/258470/10.1155/2014/258470 PMC4033417 24895560

[37] Verma Akanksha, Dixit Priyanka. Knowledge and Practices of Exclusive Breastfeeding among Women in Rural Uttar Pradesh. J Neonat Biol. 2016;5:1–7.

[38] World Health Organization (WHO). Breastfeeding and COVID-19, ‏ [Internet]. 2020 [cited 2022 Jan 20]. Available from: https://www.who.int/publications/i/item/WHO-2019-nCoV-Sci_Brief-Breastfeeding-2020.1.

[39] Singh K, Khan SM, Carvajal–Aguirre L, Brodish P, Amouzou A, Moran A. The importance of skin–to–skin contact for early initiation of breastfeeding in Nigeria and Bangladesh. Journal of Global Health. 2017;7(2). Available from:https://www.ncbi.nlm.nih.gov/pmc/articles/PMC5804505/10.7189/jogh.07.020505 PMC5804505 29423182

[40] Abdulghani N, Edvardsson K, Amir LH. Worldwide prevalence of mother-infant skin-to-skin contact after vaginal birth: A systematic review. PLOS ONE. 2018;13(10). Available from:https://www.ncbi.nlm.nih.gov/pmc/articles/PMC6209188/10.1371/journal.pone.0205696 PMC6209188 30379859

