# Correction: Assessment of knowledge and practices of exclusive breastfeeding among rural women during the COVID-19 pandemic in Egypt: a cross sectional study

**DOI:** 10.1186/s12905-025-03559-9

**Published:** 2025-01-22

**Authors:** Noura El-Gamel, Amina El-Nemer

**Affiliations:** Woman’ s Health and Midwifery Nursing, Faculty of Nursing, Mansoura University, Mansoura, Egypt

**Correction: BMC Women’s Health (2023) 23:673**

10.1186/s12905-023-02831-0

The original publication of this article [1], contained an incorrect affiliation for Noura El‑Gamel; both authors were affiliated with the same affiliation. The incorrect and correct information is listed in this correction article; the original article has been updated.

Incorrect:

Noura El‑Gamel

• Woman’s Health and Midwifery Nursing, Faculty of Nursing, Mansoura University, Damietta, Egypt

Correct:

Noura El‑Gamel

• Woman’s Health and Midwifery Nursing ,Faculty of Nursing, Mansoura University, Mansoura, Egypt
